# Safety, efficacy, and cost-effectiveness of intraoperative blood salvage in OPCABG with different amount of bleeding: a single-center, retrospective study

**DOI:** 10.1186/s13019-018-0794-6

**Published:** 2018-10-17

**Authors:** Huan Wang, Weijian Zheng, Weiping Fang, Gaige Meng, Lei Zhang, Yannan Zhou, Erwei Gu, Xuesheng Liu

**Affiliations:** Department of Anesthesiology, the First Affiliated Hospital of Anhui Medical University, No. 218 Jixi Road, Hefei, Anhui 230022 People’s Republic of China

**Keywords:** OPCABG, Autologous blood transfusion, Cost-effectiveness

## Abstract

**Background:**

We sought to evaluate the safety, efficacy, and cost-effectiveness of intraoperative blood salvage (IBS) in off-pump coronary artery bypass grafting (OPCABG) surgery with different amount of bleeding.

**Methods:**

We retrospectively reviewed the medical records of 321 patients who underwent OPCABG between December 2012 and December 2016 at our hospital. Patients treated with IBS or allogeneic blood (AB) transfusions were divided into three groups depending on the amount of bleeding respectively: IBS1 or AB1 group (400–600 ml); IBS2 or AB2 group (600–1000 ml); IBS3 or AB3 group (1000–1500 ml). The intraoperative and postoperative conditions, blood transfusion volume, clinical and hematological outcomes, and total blood transfusion cost were examined.

**Results:**

The amount of allogeneic red blood cell (RBC) transfusion in the IBSs groups were significantly lower than that in the ABs groups (*P* < 0.01). Furthermore, drainage volume 24 h post-surgery (*P* < 0.05) and white blood cell count (WBC) 2 day post-surgery (*P* < 0.01) in IBS3 group were significantly higher compared with the AB3 group. Additionally, when IBS cost was 230 USD per set, the total blood transfusion cost in the IBSs groups was significantly higher than that in the ABs groups (*P* < 0.01); however, when 199 or 184 USD, only the IBS1 group, rather than IBS2 or IBS3, showed significantly higher cost of the total blood transfusion compared with the AB1 group (*P* < 0.05).

**Conclusions:**

When the amount of bleeding was 600–1000 ml, IBS can significantly reduce the demand for allogeneic blood, and has no direct adverse effects on coagulation function and recuperation, and is cost-effective in OPCABG.

## Background

Increased intraoperative blood loss has been shown in off-pump coronary artery bypass grafting (OPCABG) due to the fact that patients with a high risk of bleeding try to receive surgery [1–3]. Despite the progress in the safety of allogeneic blood (AB) transfusions, there are still some potential risks such as immunological reactions, transfusion-transmitted infection, and bacteria contamination [4, 5]. Intraoperative blood salvage (IBS), also known as autologous blood transfusion or cell salvage, is a medical procedure of collecting, filtering and washing blood gathered from the surgical field to produce autologous blood for transfusion back to the patient [1]. In order to reduce the incidence of adverse events resulting from AB transfusion and to cope with increasing blood shortage and medical costs, IBS has been recently applied in the field of cardiac surgery.

With the progress in surgical technique and the application of advanced equipment, on one hand, the amount of intraoperative bleeding was obviously reduced due to the development of minimally invasive surgery [6]; and on the other hand, the amount of intraoperative blood loss is still fluctuating as many patients with high bleeding risk also began to undergo surgical treatment. The cost-effectiveness of IBS differs with the amount of bleeding. Until now, few data evaluating IBS in high-bleeding-risk cardiac surgery has published [7].

Thus, the aim of this study was to evaluate the safety, efficacy, and cost-effectiveness of IBS in OPCABG surgery with different amount of bleeding. To address this, we retrospectively analyzed the medical records of 321 patients who underwent OPCABG at our hospital. Patients treated with IBS or AB transfusions were divided into three groups depending on the amount of bleeding respectively. The intraoperative and postoperative conditions, blood transfusion volume, clinical and hematological outcomes, and total blood transfusion cost were examined.

## Methods

### Subjects

We retrospectively reviewed the medical records of 321 patients undergoing elective OPCABG surgery under general anesthesia from December 2012 to December 2016 in the First Affiliated Hospital of Anhui Medical University. Of all, there were 206 males and 115 females, with age of 50–75 years, body mass index (BMI) of 16–34, and the American Society of Anesthesiologists’ (ASA) physical status of II-IV level. A previous study has indicated that the application of IBS is more meaningful in patients with more than 1000 ml blood loss during surgery [1]^.^ Nevertheless, at present, the intraoperative blood loss is usually less than 1000 ml, and Advanced Trauma Life Support System (ATLS) [8] believe that human conditions are stable only when the blood loss was less than 15% (about 600 ml when calculating with a systemic blood volume of 4000 ml). In clinical applications, we found that when the bleeding volume was less than 600 ml, Cell Saver, in most cases, washed out autologous red blood cells with lower hematocrit, and the application efficiency was poor; when the bleeding volume was less than 400 ml, blood transfusion was usually not needed. Therefore, for patients who underwent IBS, they were divided into IBS1 group (bleeding volume 400–600 ml), IBS2 group (600–1000 ml), IBS3 group (1000–1500 ml) depending on the amount of intraoperative blood loss. Patients with allogeneic blood transfusions blood were divided into AB1 group (bleeding volume 400–600 ml), AB2 group (600–1000 ml), AB3 group (1000–1500 ml).

The exclusion criteria were as follows: (1) cases of abandoning treatment and self-discharge due to the personal wishes of patients and their families; (2) cases of secondary surgery; (3) cases of severe data loss; (4) cases in which the amount of intraoperative blood loss failed to meet the criteria.

**Fig. 1:**
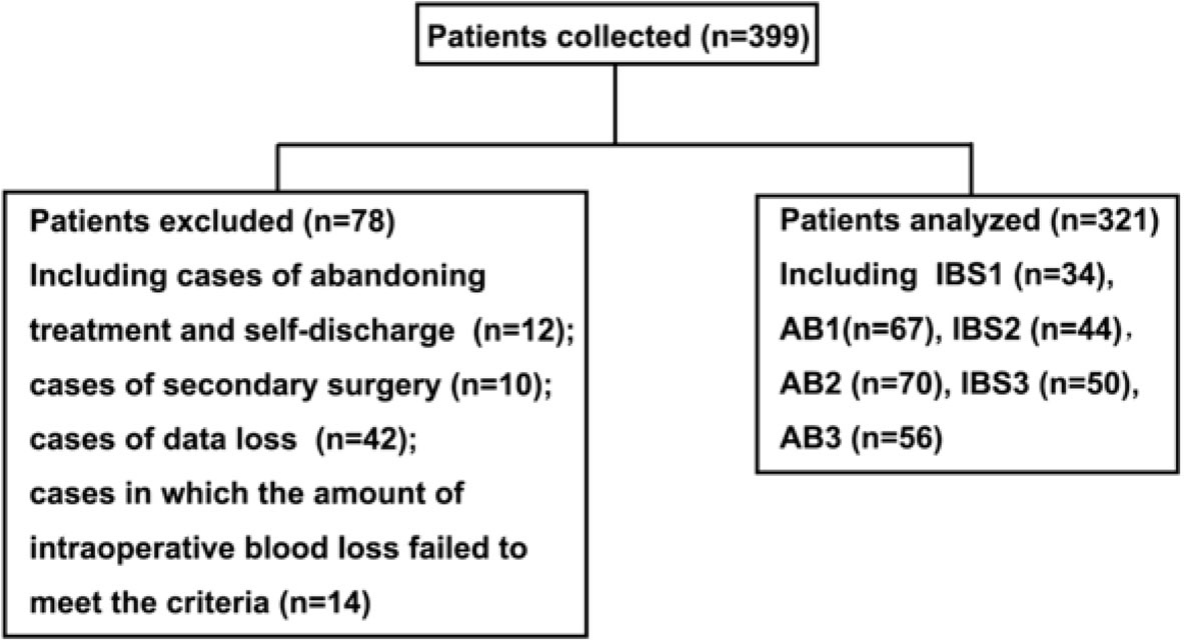
CONSORT diagram of the flow of patients

The study was approved by the Ethics Committee of the First Affiliated Hospital of Anhui Medical University. Written informed consent was obtained from each patient. The CONSORT diagram of the flow of patients was summarized in Fig. 1.

### Surgery and blood transfusion

All patients received intratracheal intubation and combined intravenous with inhalation general anesthesia in each case, the red blood cell salvage was performed using a Cell Saver 5 System (Haemonetics; Braintree, MA). Normal saline (500 ml) containing 15,000 μl heparin was used as the anticoagulant solution. The blood reservoir and tubing are pre-impregnated with 150 ml anticoagulant solution before the start of blood recovery. The drip rate was 1 to 2 per second depending on the speed of blood flow during machine operation. After filtrated, centrifugated, washed, the recovered blood became autologous blood (autologous RBC) with a hematocrit of 50% to 60% [9, 10], and was then transfused back to the patients immediately. All autologous RBC was transfused back before the end of surgery. Blood transfusions were administrated if hemoglobin (HGB) level < 9 g per deciliter. HBG level was maintained at ≥10 g per deciliter [11]. In order to prevent coagulation dysfunction, fresh frozen plasma and platelets were transfused according to thromboelastogram data and the clinician experience when necessary.

### Charges

The costs in the ABs group are the sum of the costs of all infusion of allogeneic blood products, including allogeneic red blood cells, fresh frozen plasma and other costs. Each unit of allogeneic red blood cells was 34 USD; fresh frozen plasma per 100 ml was 6 USD; Each dose of therapeutic platelets was 218 USD; Each unit of cold precipitation was 15 USD.

The costs in the IBSs group include cell saver consumables usage fees and allogeneic blood products costs. Each set of consumables includes a collection reservoir, a combination line, a centrifuge cup, a reinfusion bag, and a waste bag. Due to the reduction of costs, the cost of cell saver consumables has been declined year by year. Here, the cost of cell saver was divided into three grades, 230, 199 and 184 USD, which were due to the different prices of cell saver consumables in different periods. Patients in the IBSs group were treated with a recovery autologous blood transfusion technique, which only charged the consumables usage fee, and did not charge the technical and labor costs of the IBS. If a patient in the IBS group receives an allogeneic blood product, it is also charged according to the cost of the allogeneic blood products.

### Data collection

The main indicators included intraoperative blood loss, hemoglobin (HGB), white blood cell count (WBC), RBC, platelet (PLT) values before and 1, 2, and 7 days after surgery, infusion of allogeneic RBC, PLT, and plasma, and total transfusion costs. The minor indicators included mechanical ventilation duration, intensive care unit (ICU) stay, hospital stay, etc. The intraoperative blood loss is estimated by calculating the sum of the blood in the vacuum aspirator, the blood in the autoblood reservoir, the gauze, and the blood in the cotton pad.

### Statistical analysis

All statistical analyses were performed using SPSS statistical software package standard version 17.0 (SPSS, Inc., Chicago, IL, USA). Normally distributed data are expressed as the mean ± standard deviation (SD). Two independent sample *t*-tests were used for comparison between groups. χ^2^-tests were used for comparison of counting data. *P* < 0.05 was considered to indicate a statistically significant difference.

## Results

### General conditions of all subjects

As shown in Table 1, there was no significant difference in gender, age, ASA status, cardiac function, and complications including hypertension and diabetes of all the 321 participants.

**Table 1:** Comparison of general data

Group	N	Male/Female (N)	Age	BMI (kg/m^2^)	ASA II/III/IV	Cardiac function II/III/IV	Hypertension (%)	Diabetes (%)
IBS1	34	21/13	64.82 ± 6.25	23.31 ± 3.36	13/20/1	14/19/1	18 (53)	8 (24)
AB1	67	34/33	62.73 ± 7.04	23.76 ± 3.45	22/43/2	24/41/2	25 (40)	14 (22)
IBS2	44	24/20	62.82 ± 9.45	23.79 ± 3.75	12/32/0	18/26/0	24 (55)	9 (21)
AB2	70	45/25	63.97 ± 6.48	22.96 ± 3.37	17/52/1	25/44/1	42 (60)	13 (19)
IBS3	50	33/17	65.22 ± 8.91	24.32 ± 3.67	10/37/3	16/31/3	30 (60)	11 (22)
AB3	56	39/17	63.41 ± 7.96	23.12 ± 3.39	11/43/2	18/36/2	27 (48)	14 (25)

*BMI* body mass index, *ASA* the American Society of Anesthesiologists’ physical status

### Comparison of intraoperative and postoperative conditions as well as blood transfusions

As shown in Tables 2 and 3, there were no significant differences between the ABs and IBSs groups in terms of intraoperative and postoperative conditions in patients, including the number of intra-aortic balloon pump counterpulsation (IABP), number of bridges, operation duration, hospital stay, ICU stay, mechanical ventilation duration, Intraoperative blood loss, total platelet transfusion at therapeutic dose, and total plasma transfusion. As indicated in Table 3, the drainage volume 24 h post-surgery in the IBS3 group was significantly higher than that in the AB3 group (*P* < 0.05). Furthermore, the total infusion of allogeneic red blood cells in the IBSs groups was significantly lower than that in the corresponding ABs groups (*P* < 0.01).

**Table 2:** Comparison of intraoperative and postoperative conditions in patients

Group	N	IABP (%)	Number of bridges	Operation duration (min)	Hospital stay (day)	ICU stay (h)	Mechanical ventilation duration (h)
IBS1	34	9 (27)	3.44 ± 1.23	225.41 ± 47.45	26.88 ± 9.17	55.35 ± 62.97	13.09 ± 4.94
AB1	67	28 (44)	3.25 ± 0.84	239.87 ± 32.46	28.35 ± 9.03	62.97 ± 30.24	13.71 ± 5.07
IBS2	44	15 (34)	3.66 ± 0.88	272.55 ± 47.71	28.45 ± 7.89	70.20 ± 35.08	14.75 ± 5.28
AB2	70	22 (31)	3.47 ± 1.05	259.50 ± 47.70	27.00 ± 8.14	70.00 ± 38.29	13.91 ± 5.94
IBS3	50	23 (46)	3.74 ± 0.77	283.00 ± 50.02	27.54 ± 8.10	73.66 ± 34.30	15.98 ± 4.51
AB3	56	20 (36)	3.63 ± 1.03	284.52 ± 38.15	27.34 ± 8.56	67.34 ± 38.87	15.32 ± 8.00

*IABP* intra-aortic balloon pump counterpulsation, *ICU* intensive care unit

**Table 3:** Comparison of bleeding and blood transfusion in patients

Group	N	Intraoperative blood loss (ml)	Drainage volume 24 h post-surgery (ml)	Allogeneic red blood cell transfusion (U)	Total platelet transfusion (therapeutic dose)	Total plasma transfusion (ml)
IBS1	34	476.94 ± 96.75	470.15 ± 154.01	1.97 ± 1.98^b^	0.00 ± 0.00	426.47 ± 260.31
AB1	67	447.49 ± 110.86	469.08 ± 174.37	4.7 ± 1.73	0.02 ± 0.13	518.36 ± 291.45
IBS2	44	796.05 ± 99.58	474.95 ± 129.14	1.86 ± 2.73^b^	0.02 ± 0.15	479.55 ± 383.72
AB2	70	789.77 ± 100.22	511.57 ± 170.90	5.19 ± 2.76	0.06 ± 0.23	596.43 ± 404.35
IBS3	50	1393.26 ± 270.87	626.16 ± 189.38^a^	3.78 ± 4.60^b^	0.08 ± 0.27	738.00 ± 688.87
AB3	56	1331.80 ± 276.39	543.32 ± 185.71	6.52 ± 4.11	0.07 ± 0.26	882.14 ± 465.87

^a^*P* < 0.05,^b^*P* < 0.01 vs. AB group

### Comparison of hematological outcomes

Next, we compared hematological outcomes of all the patients. Data revealed that the white blood cell count (WBC) 2 day post-surgery in the IBS3 group was significantly higher compared with the AB3 group (Table 4, *P* < 0.01).

**Table 4:** Comparison of hematological outcomes

Indexes	Group	N	Pre-surgery	1 d post-surgery	2 d post-surgery	7 d post-surgery
HGB(g/L)	IBS1	34	126.41 ± 12.03	123.41 ± 15.51	119.24 ± 16.86	124.65 ± 19.05
	AB1	67	127.57 ± 11.38	124.90 ± 12.48	119.24 ± 12.30	127.81 ± 73.53
	IBS2	44	131.43 ± 11.78	123.86 ± 13.98	118.55 ± 17.12	125.30 ± 17.25
	AB2	70	129.97 ± 13.09	124.57 ± 13.21	120.11 ± 14.66	130.17 ± 16.72
	IBS3	50	134.54 ± 13.16	115.62 ± 19.41	113.22 ± 14.18	126.18 ± 14.13
	AB3	56	137.14 ± 13.07	118.71 ± 13.02	116.91 ± 15.12	129.32 ± 16.03
WBC(10^9^/L)	IBS1	34	6.39 ± 1.43	14.43 ± 4.00	13.99 ± 4.54	8.99 ± 2.42
	AB1	67	6.18 ± 1.64	13.98 ± 3.67	12.98 ± 4.29	9.62 ± 2.62
	IBS2	44	6.59 ± 1.49	15.52 ± 4.43	14.54 ± 3.70	9.45 ± 2.83
	AB2	70	6.73 ± 1.57	14.52 ± 3.59	13.17 ± 4.66	9.97 ± 2.82
	IBS3	50	6.62 ± 1.76	14.06 ± 3.92	15.08 ± 5.07^b^	10.63 ± 4.45
	AB3	56	6.83 ± 1.78	13.53 ± 3.55	12.53 ± 3.53	9.79 ± 2.77
RBC(10^12^/L)	IBS1	34	4.17 ± 0.53	4.06 ± 0.44	3.91 ± 0.50	4.11 ± 0.55
	AB1	67	4.23 ± 0.38	4.16 ± 0.41	3.94 ± 0.43	4.20 ± 0.53
	IBS2	44	4.29 ± 0.48	4.07 ± 0.48	3.87 ± 0.56	4.19 ± 0.64
	AB2	70	4.30 ± 0.45	4.08 ± 0.39	3.92 ± 0.47	4.21 ± 0.53
	IBS3	50	4.36 ± 0.45	3.80 ± 0.39	3.56 ± 0.39	4.10 ± 0.41
	AB3	56	4.51 ± 0.41	3.97 ± 0.43	3.65 ± 0.51	4.18 ± 0.60
PLT(10^9^/L)	IBS1	34	200.15 ± 65.35	191.68 ± 72.16	152.76 ± 58.65	209.12 ± 65.99
	AB1	67	213.25 ± 65.21	167.84 ± 55.63	135.76 ± 73.53	203.24 ± 73.53
	IBS2	44	212.45 ± 57.45	184.39 ± 61.64	156.68 ± 69.92	212.30 ± 97.22
	AB2	70	219.91 ± 71.88	184.43 ± 55.13	150.47 ± 54.44	220.09 ± 80.20
	IBS3	50	212.26 ± 50.83	172.58 ± 57.43	142.82 ± 52.47	236.44 ± 97.12
	AB3	56	208.68 ± 61.78	156.20 ± 38.59	137.63 ± 47.12	213.23 ± 91.22

*HGB* hemoglobin, *WBC* white blood cell count, *RBC* red blood cell count, *PLT* platelet

^b^*P* < 0.01 vs. AB group

### Comparison of total blood transfusion cost

As reflected in Table 5, the total blood transfusion cost in the IBSs groups was significantly higher than that in the ABs groups when the IBS cost was 230 USD per set (*P* < 0.05). However, when 199 or 184 USD per set, only the IBS1, rather than IBS2 or IBS3 group, showed significantly higher cost of the total blood transfusion compared with the AB1 group (*P* < 0.05).

**Table 5:** Comparison of the total blood transfusion cost

Group	N	Total blood transfusion cost (USD)		
		230	199	184
IBS1	34	327.5 ± 96.9 ^a^	297.0 ± 96.9 ^a^	281.7 ± 96.9 ^a^
AB1	67	233.7 ± 107.9	233.7 ± 107.9	233.7 ± 107.9
IBS2	44	333.8 ± 145.1 ^a^	303.2 ± 145.1	287.9 ± 145.1
AB2	70	260.9 ± 157.5	260.9 ± 157.5	260.9 ± 157.5
IBS3	50	439.2 ± 276.2 ^a^	408.6 ± 276.2	393.3 ± 276.2
AB3	56	332.3 ± 219.8	332.3 ± 219.8	332.3 ± 219.8

^a^*P* < 0.05 vs. AB group

## Discussion

Previous studies have shown that infusion of allogeneic blood has adverse effects on the long-term prognosis of patients [12, 13], especially for patients with existing cardiac problems, which can increase the mortality within 5 years after surgery [14]. At the same time, autologous blood transfusion has been recently used in the field of cardiac surgery due to the tight blood supply. However, the blind application of IBS technology will not only fail to exert its advantages, but also increase hospitalization costs and increase the burden on patients. Accordingly, in this study, a single OPCABG surgery was conducted, and bleeding was divided into three different hemorrhage layers to evaluate the impact of IBS on outcomes following OPCABG surgery.

The results showed that when the blood loss ranged from 600 to 1000 ml, the application of IBS led to a significant reduction in the transfusion volume of allogeneic blood. Meanwhile, IBS maintained the HGB and RBC values similar to that of the ABs groups. Moreover, IBS did not increase the number of postoperative leukocytes and drainage volume 24 h post-surgery. Additionally, IBS did not affect the mechanical ventilation duration, ICU stay, hospital stay, and total transfusion cost, showing the best cost-effectiveness.

Our results also revealed that, when the amount of blood loss ranged from 400 to 600 ml, IBS could reduce the total allogeneic RBC infusion to a certain degree. We also found that IBS significantly increased the total transfusion cost compared with the transfusion of allogeneic blood only. In addition, the current standards for blood transfusion for cardiac patients differs depending on the specific conditions, and the comparison of restrictive and loose blood transfusion standards are still to be studied [15]. Therefore, whether or not a patient with less than 600 ml blood loss applies IBS can depend on the condition of the patient and the experience of clinicians.

When the blood loss ranged from 1000 to 1500 ml, the IBS3 group showed higher 24 h post-surgery drainage volume compared with the AB3 group but there was no significant difference in the number of postoperative platelets counts between the two groups. This may be attributed to less infusion of fresh frozen plasma and thus lack of coagulation factors. The results also showed that the IBSs groups had lower plasma transfusion volume than in the ABs groups, indicating that the application of the IBS technique requires a reasonable amount of infusion of blood products such as fresh frozen plasma rich in coagulation factors when the amount of intraoperative blood loss is large, otherwise it may affect the coagulation function of patients. This is consistent with the results of Shen et al. [16]. The cause of this phenomenon may also include an increase in autologous blood transfusion, which dilutes blood coagulation factors and platelets, increases the heparin content that enters the body [17], thereby promoting damage to the coagulation system. Furthermore, the number of white blood cells in the IBS3 group was higher than that in the AB3 group on the second postoperative day. In addition, we found the WBC value 2 day post-surgery was in the IBS3 group was significantly higher compared with the AB3 group which is different from the findings of Cui et al. [18]. The different results may be attributed to several factors, including less blood loss (700 ± 50 ml), and reduced blood transfusion volume, and the different type of surgery in the study by Cui et al. Currently used autologous blood transfusion apparatus can destroy cell components during blood recovery and centrifugation, activate platelets and leukocytes. Conventional washing fails to completely remove activated leukocytes, so reinfusion into patients can promote the release of inflammatory mediators and further aggravated the systemic inflammatory response. Clinically, WBC is widely used for the auxiliary diagnosis of infection, and the degree of WBC elevation is directly related to the degree of trauma [19]. IBS may retain more white blood cells and inflammatory mediators compared with allogeneic blood products with leukocyte-depleted red blood cells. Therefore, the addition of a leukocyte filter would be more beneficial to patients when using IBS in high-bleeding-risk cardiac surgery [20].

Moreover, we also observed that the total blood transfusion cost in the IBSs groups was significantly higher than that in the ABs groups when the IBS cost was 230 USD per set. However, when 199 or 184 USD per set, the IBS1 group showed significantly higher cost of the total blood transfusion compared with the AB1 group. With the reduction of IBS charges and the increase of the blood loss amount, the cost-effectiveness of IBS has gradually emerged. Xie et al. [21] demonstrated that the use of IBS in OPCABG surgery reduced the chances of patients exposure to allogeneic blood, decreased the incidence of transfusion-related diseases and transfusion reactions, but it increased the total transfusion costs. Attaran et al. [22] also stated that the routine use of IBS is not cost-effective. Malhotra *et al.* [1] proposed that the application of IBS is more meaningful in patients with more than 1000 ml blood loss during surgery. Wang et al. [23] reported that a cell saver may be beneficial only when it is used for shed blood and/or residual blood or during the entire operative period. Processing cardiotomy suction blood with a cell saver only during cardiopulmonary bypass has no significant effect on blood conservation and increases fresh frozen plasma transfusion. These abovementioned findings are s not exactly the same as our findings. This may be attributed to the large difference in charges for IBS and allogenic blood products between at home and abroad, as well as the higher charge of IBS and the low cost of allogeneic blood products at home. In addition, the decrease in intraoperative blood loss or inappropriate use of blood transfusion apparatus results in less autologous blood recovery and even failure to wash autologous blood, which may also affect the cost. Moreover, the limited sample size in this study should also be noted. We propose that the results of cost-benefit analysis may differ when the bleeding amount is higher or the IBS charge is lower.

This study was a retrospective analysis, in which patients in the IBS group were started to use autologous blood transfusions immediately after surgery. Due to the inability to predict intraoperative blood loss, we used the commonly used loose blood transfusion standard. That is, when the patient’s HGB value was < 9 g/L, blood transfusion can be started. In addition, it is necessary to refer to the preoperative and intraoperative HGB values and the specific operation situation. According to the restrictive blood transfusion standard, patients who may have less than 600 ml of blood loss do not need blood transfusions, and thus do not need to apply IBS. This is the reason why this paper analyzes the cost-effectiveness of stratified blood loss analysis.

## Conclusions

In conclusion, IBS has different efficacy in different bleeding situations. Particularly, when the amount of bleeding ranges from 600 to 1000 ml, IBS can significantly reduce the demand for allogeneic blood, and has no direct adverse effects on coagulation function and postoperative recovery of patients, and is cost-effective in OPCABG under the current charge standard. The role and cost-effectiveness of using IBS in patients with old age and high-bleeding risks need to be further analyzed. Furthermore, how to accurately screen high-bleeding-risk patients is still a question worthy of study. In addition, a prospective, multi-center, randomized, controlled study is also needed to clarify the best application guidelines for IBS technology so that the technology could be applied more accurately.

## Abbreviations

AB: allogeneic blood
ATLS: Advanced Trauma Life Support System
HGB: hemoglobin
IBS: intraoperative blood salvage
ICU: intensive care unit
OPCABG: off-pump coronary artery bypass grafting
PLT: platelet
RBC: red blood cell
WBC: white blood cell count
